# Hodgkin Lymphoma: An Unusual Presentation With Hemophagocytic Lymphohistiocytosis

**DOI:** 10.7759/cureus.90007

**Published:** 2025-08-13

**Authors:** Taiwo Ikuesan, Ella Fernandes, Amal Humayun

**Affiliations:** 1 General Medicine, King's College Hospital NHS Foundation Trust, Orpington, GBR

**Keywords:** abdominal lymphadenopathy, classic hodgkin lymphoma, h score, positron emission tomography (pet scans), secondary hemophagocytic lymphohistiocytosis

## Abstract

Hodgkin lymphoma (HL) can present with non-specific systemic symptoms, often mimicking infection, autoimmune disease, or post-surgical complications. When compounded by recent major surgery and ongoing inflammatory features, diagnosis can be significantly delayed. This case highlights the diagnostic complexity of HL in a postoperative setting, complicated by recurrent sepsis-like presentations, persistently raised inflammatory markers, and overlapping differentials, including graft infection and hemophagocytic lymphohistiocytosis (HLH).

A 71-year-old male underwent elective abdominal aortic aneurysm (AAA) repair, complicated by significant intraoperative blood loss. He initially recovered but began presenting with recurrent fevers, abdominal pain, vomiting, and deranged liver function tests (LFTs). He underwent comprehensive imaging, which revealed peri-graft fluid collection on CT, fluorodeoxyglucose (FDG)-avid lymphadenopathy on PET/CT, and a mobile aortic valve structure on an echocardiogram, raising concerns for a graft infection, disseminated infection of unknown origin, and infective endocarditis, respectively. Despite multiple courses of intravenous antibiotics, he continued to have persistent fever spikes, and bicytopenia coupled with hyperferritinemia prompted consideration of HLH or malignancy. Further imaging, including a liver MRI and PET imaging, revealed multiple hyperintense hepatic foci and widespread nodal uptake. With clinical deterioration, the patient was empirically treated with anakinra and steroids for HLH with an unclear underlying driver, resulting in temporary improvement. Bone marrow biopsy ultimately confirmed a diagnosis of HL.

This case highlights the importance of maintaining a broad differential when managing persistent systemic inflammation following surgery. HL should be considered when infection and autoimmune causes are excluded, especially in the presence of FDG-avid lymphadenopathy, cytopenias, and hyperferritinemia. Early hematology involvement and tissue diagnosis are key to avoiding delays in treatment.

## Introduction

Hodgkin lymphoma (HL) is a malignancy of the lymphatic system characterized by the presence of Reed-Sternberg cells, large, abnormal B lymphocytes with distinctive morphological features [1]. HL is classified into two main types: classical HL (cHL), which accounts for approximately 95% of cases, and nodular lymphocyte-predominant HL (NLPHL) [2].

Globally, HL is a relatively rare cancer, with an estimated 83,000 new cases and 23,000 deaths reported in 2020 [3]. The disease exhibits a bimodal age distribution, with incidence peaks occurring in young adults (15-35 years) and in older adults (over 55 years) [4]. HL is more prevalent in males and has been associated with risk factors such as Epstein-Barr virus (EBV) infection, immunosuppression, and a family history of lymphoma [5].

Despite its high curability, diagnostic delays in HL are not uncommon. These delays are often attributed to the non-specific nature of its initial symptoms, such as painless lymphadenopathy, fatigue, and intermittent fevers, which can mimic benign conditions [6]. Additionally, the rarity of the disease and atypical presentations such as chest pain with initial findings of pericardial effusion [7] and impending tamponade [8], sternal and rib fractures [9], and ocular inflammation [10], to name a few, can lead to misdiagnosis or delayed consideration in differential diagnoses [11].

Early and accurate diagnosis of HL is crucial, as it significantly influences treatment outcomes. Studies have demonstrated that early-stage HL has a favorable prognosis, with five-year survival rates exceeding 90% when appropriate therapy is initiated promptly [12]. Therefore, heightened clinical awareness and timely diagnostic evaluation are essential to improve patient outcomes.

Hemophagocytic lymphohistiocytosis (HLH) is a syndrome characterized by hyperinflammation and subsequent autoimmune host tissue damage, triggered by a plethora of drivers, including inborn genetic mutations, infection, and neoplastic disorders [13]. Characterised by fever, splenomegaly, cytopenia, hyperferritinemia, hypertriglyceridemia, and tissue biopsy exhibiting hemophagocytosis, it is important to consider HL when investigating underlying drivers for HLH [14].

This case illustrates the diagnostic uncertainty surrounding HL in the presence of overlapping infectious and inflammatory presentations like graft infections, sepsis, and HLH.

## Case presentation

A 71-year-old male, previously clinically well, underwent elective abdominal aortic aneurysm (AAA) repair, complicated by massive intraoperative blood loss requiring transfusion. He was admitted to intensive care, stepped down to the ward, and discharged on antibiotics for a urinary tract infection, with no immediate postoperative concerns. The patient presented in the hospital on four separate occasions (Table 1) for blood results across these admissions.

**Table 1:** Blood results from the first three months post-operation and the latest presentation to the hospital HB: hemoglobin; WCC: white cell count; CRP: C-reactive protein; PT: prothrombin time; APTT: activated partial thromboplastin time; ALT: alanine transaminase; AST: aspartate transferase; ALP: alkaline phosphatase; GGT: gamma-glutamyl transferase; LDH: lactate dehydrogenase * Blood tests not carried out during the period mentioned

Tests	1st month post-op		2nd month post-op		3rd month post-op		Final presentation leading to diagnosis			
	Admission	Discharge	Admission	Discharge	Admission	Discharge	Admission	Week 1	Week 2 (Anakinra started)	Week 3
HB (125-170 g/L)	106	95	84	104	101	90	93	84	85	77
WCC (2.9-9.6 x 10^9^/L)	4.6	5.2	2.6	3.6	4.1	2.7	3.3	2.5	5.3	3.1
Platelet (140-400 x 10^9^/L)	221	361	211	292	150	286	293	256	187	120
CRP (<5mg/L)	226	26	140	15	214	134	216	139	119	111
PT (9.7-12.0 sec)	13.2	*	12.8	*	12.6	12.4	13.5	13.1	*	12.6
APTT (20.0-20.9 sec)	30.8	*	38.1	*	35.3	*	34.5	40.6	*	42/5
Fibrinogen (1.70-4.90 g/L)	*	*	*	*	5.40	*	5.49	5.01	5.01	4.14
Total bilirubin (<21 umol/L)	19	11	11	36	44	49	65	64	79	86
ALT (0-45 U/L)	*	*	*	*	*	*	74	*	*	*
AST (5-34 U/L)	*	*	125	103	*	*	135	149	54	96
ALP (30-130 U/L)	360	776	1144	1449	1006	1881	1694	2533	1373	2037
GGT (<55 U/L)	192	508	771	1325	740	621	547	660	342	619
Ferritin (15-200 ug/L)	*	*	4175	2266	*	*	6184	8103	9174	7071
Triglycerides (mmol/L)	*	*	*	*	*	*	*	1.7	2.2	1.8
LDH (125-220 U/L)	*	*	*	249	153	*	199	*	134	227

First month post-op

**Figure 1:**
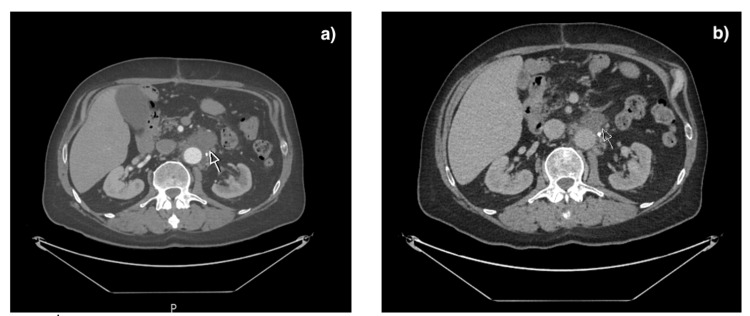
Computed tomography (CT) scan of abdomen and pelvis a: postoperative peri-graft collection (arrow); b: improvement in peri-graft collection three months postoperatively (arrow).

He presented with a three-day history of fever, vomiting, and generalized abdominal pain. Inflammatory markers were raised, and a CT abdomen-pelvis revealed a collection abutting the proximal graft anteriorly with subtle peripheral enhancement, raising concerns of graft infection (Figure 1a). However, the vascular team deemed the findings consistent with postoperative changes. Liver enzymes were elevated; ultrasound suggested mild reactive steatosis. He improved on IV antibiotics and was discharged.

Two months post-op

He returned with vomiting, night sweats, and shivering. Inflammatory markers were again elevated. A CT angiogram showed peri-aortic fat stranding; a PET scan revealed a small post-op fluid collection near the graft and widespread fluorodeoxyglucose (FDG)-avid lymphadenopathy, suggestive of disseminated infection. A PET scan revealed a small post-op fluid collection near the graft and widespread FDG-avid lymphadenopathy with hepatic, splenic, and skeletal foci. The findings were highly suspicious for disseminated infection, and lymph node sampling for microscopy, culture, and sensitivities was suggested if blood cultures remained negative. A decision was made to stop antibiotics to reassess clinically, and he remained stable. Magnetic resonance cholangiopancreatography (MRCP) was normal despite persistently deranged liver function tests (LFTs).

Three months post-op

He re-presented with left lower flank pain, fever, dark urine, and vomiting. Sepsis of unknown origin was suspected; blood cultures remained negative. Anemia and leukopenia were noted. Repeat CT showed improving peri-graft collection (Figure 1b). He improved with IV antibiotics and was discharged.

Subsequent presentation

**Figure 2:**
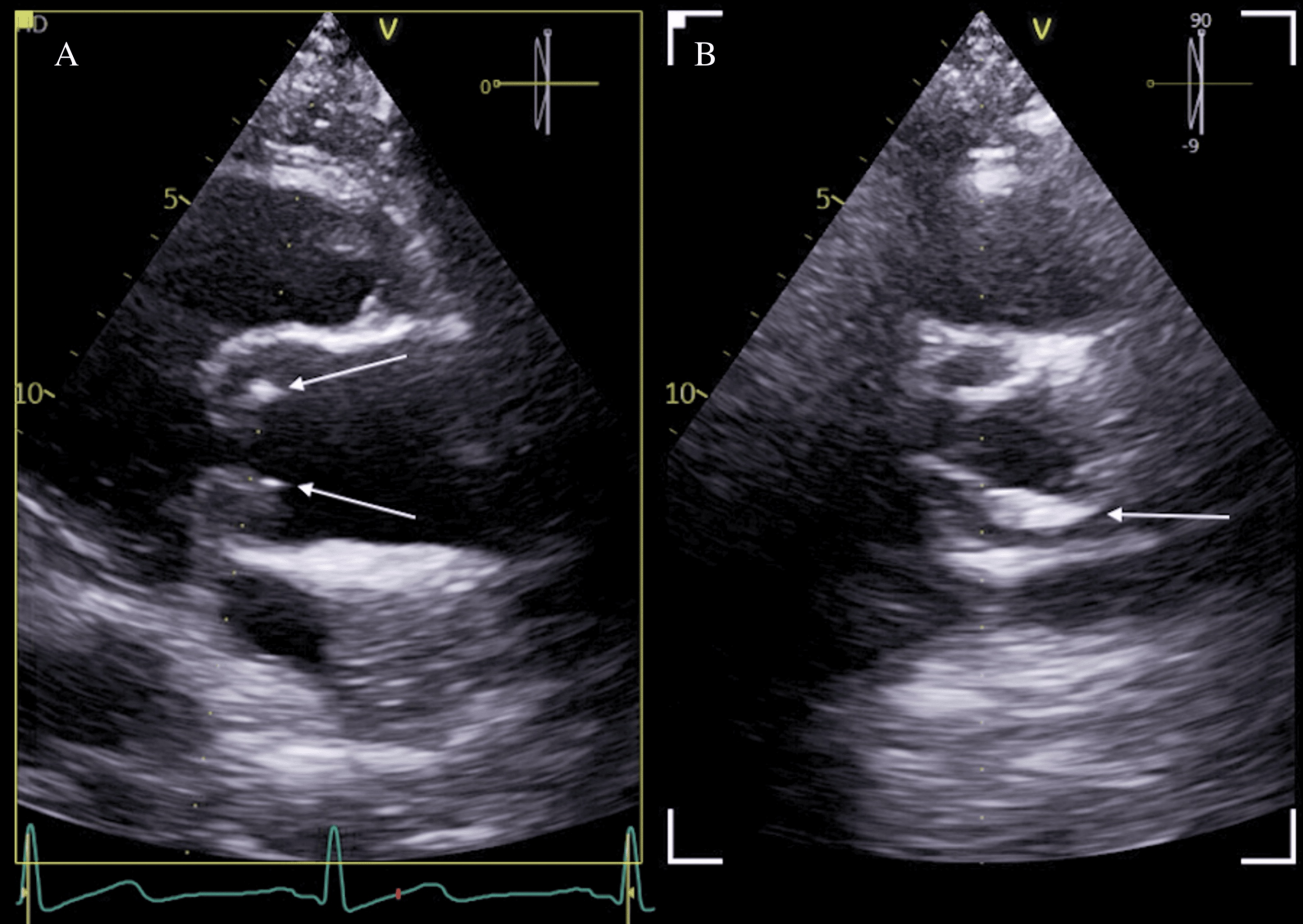
Transthoracic echocardiogram (TTE) findings Arrows in A and B point to a small mobile structure on the aortic valve, but are not suggestive of vegetation.

Shortly after, he presented again with epigastric pain, fever, and vomiting. Suspected causes included graft infection vs. infective endocarditis. Transthoracic echocardiogram (TTE) showed a mobile aortic valve structure (Figure 2), but infective endocarditis (IE) was deemed unlikely. He had persistent deranged LFTs and bicytopenia. Hematology considered anemia of chronic disease and neutropenia secondary to sepsis or drug reaction. Blood cultures were negative; ferritin was markedly elevated, prompting consideration of HLH or malignancy. Lactate dehydrogenase (LDH) was notably normal.

**Figure 3:**
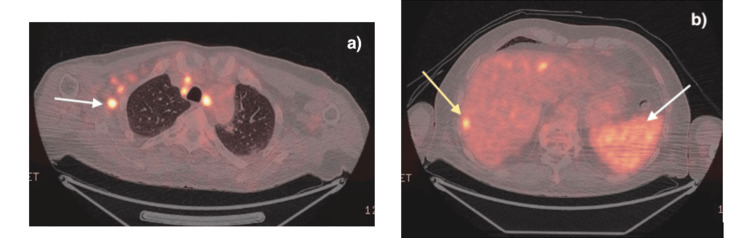
Positron emission tomography (PET) scan showing fludeoxyglucose (FDG) avid lymphadenopathy with hepatic and splenic uptake a: lymphadenopathy (white arrow); b: splenic uptake (white arrow), and hepatic uptake (yellow arrow).

**Figure 4:**
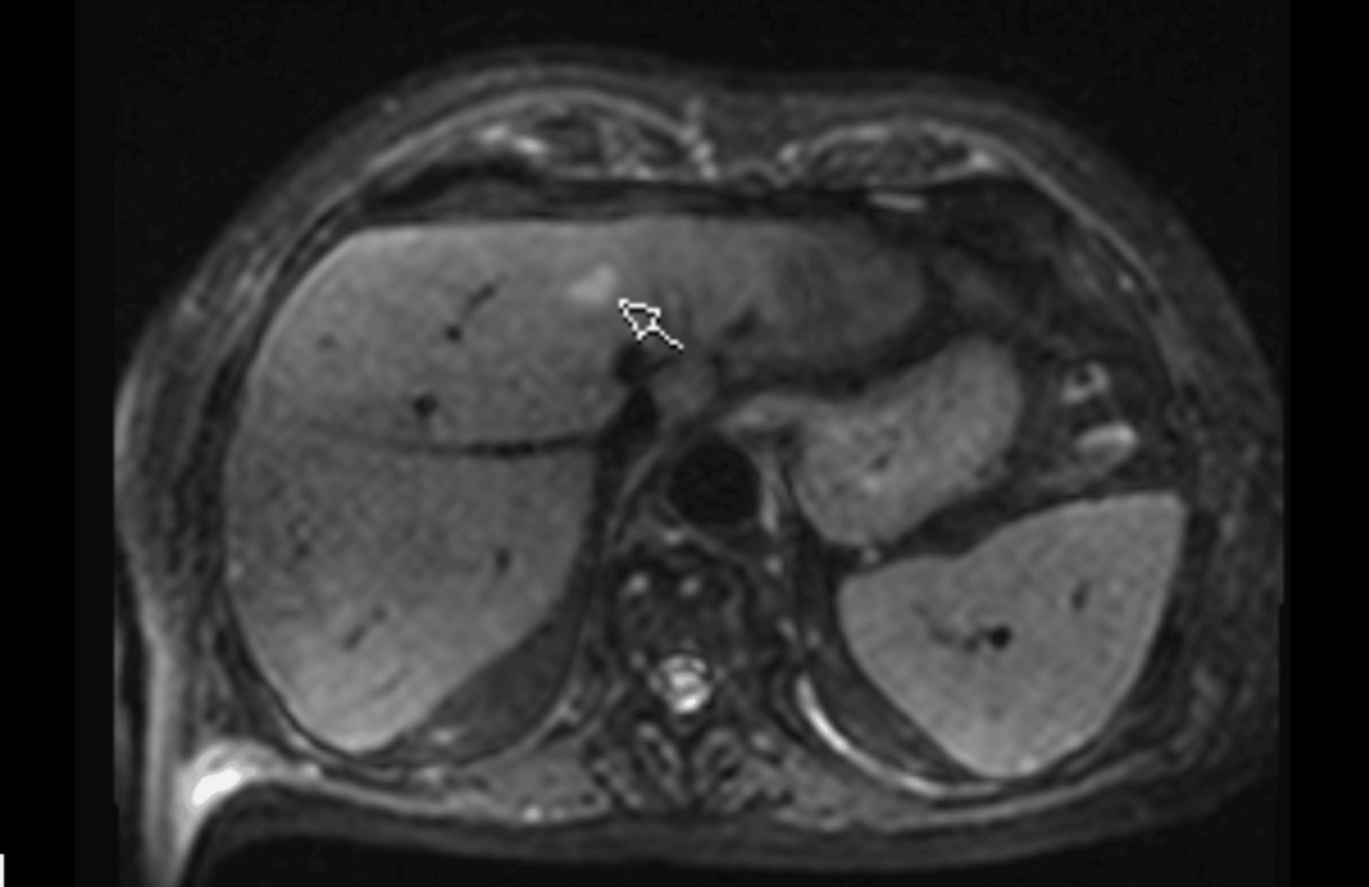
Magnetic resonance imaging (MRI) of the liver The arrow points to one of several hyperintense foci in the liver.

The repeat PET echoed previous findings of widespread FDG-avid lymphadenopathy with hepatic and splenic uptake (Figure 3), and in conjunction with raised alkaline phosphatase (ALP) and ongoing fever spikes with no clear infectious source, further consolidated the suspicion of HLH with an unclear underlying driver. The liver MRI showed multiple hyperintense foci, which, in conjunction with nodal involvement, suggested possible lymphomatous infiltration (Figure 4).

While awaiting bone marrow and lymph node biopsies, the patient clinically deteriorated with worsening cytopenia, splenomegaly, and rising ferritin levels, resulting in an H-score of 193, indicating an 80-88% probability of HLH. The H-score is a diagnostic tool for HLH that takes into consideration fever, organomegaly, number of cytopenias, ferritin levels, triglycerides, fibrinogen, serum aspartate aminotransferase (AST), features of hemophagocytosis on bone marrow (BM) aspirate, and known underlying immunosuppression. In view of this, he was started on anakinra and steroids, which led to a temporary clinical improvement. The bone marrow and lymph node biopsy results came shortly after, which confirmed HL as the underlying driver for HLH. The patient commenced chemotherapy immediately after diagnosis, and months-long pyrexia that was unresponsive to antibiotics, interleukin antagonists, and steroids. The regimen was doxorubicin, vinblastine, dacarbazine, and brentuximab vedotin (BV AVD). The symptoms resolved within a few days of starting treatment.

## Discussion

HL is a distinct type of B-cell lymphoid malignancy characterized by the presence of neoplastic Hodgkin and Reed-Sternberg (HRS) cells within an extensive inflammatory microenvironment [15]. HL is classified into two entities: cHL, which accounts for the majority of cases, and NLPHL, each exhibiting unique histopathological and clinical features [16].

The diagnosis of HL is established through histological examination of involved lymphoid tissue, revealing characteristic HRS cells in cHL or "popcorn" cells in NLPHL. Immunophenotyping is essential, with cHL typically expressing CD30 and CD15, while NLPHL expresses B-cell markers such as CD20 [17]. Staging is performed using the Ann Arbor system, often supplemented with PET scans to assess disease extent and guide treatment [18].

Pathophysiologically, HL arises from germinal center B cells that have evaded apoptosis, leading to the formation of HRS cells. These cells exhibit aberrant activation of signaling pathways, including NF-κB and JAK/STAT, contributing to their survival and proliferation. Additionally, the tumor microenvironment plays a pivotal role, with HRS cells secreting cytokines and chemokines that recruit various immune cells, creating an immunosuppressive milieu that facilitates tumor growth [19].

HL typically presents with painless lymphadenopathy, most commonly in supradiaphragmatic regions such as the cervical, supraclavicular, anterior mediastinal, and axillary lymph nodes [20]. Less frequently, nodal involvement may extend to splenic, abdominal, hilar, and inguinofemoral areas. Approximately 30% of patients experience B symptoms, which include fever above 38 degrees C, night sweats, and unexplained weight loss exceeding 10% over six months. Mass effect from enlarged lymph nodes may cause symptoms such as shortness of breath, chest or back pain, and facial edema due to superior vena cava syndrome. Although extranodal involvement is uncommon, it may affect the spleen, bone marrow, bones, lungs, or liver. Other notable features include chronic or severe pruritus and lymph node pain triggered by alcohol consumption [21]. 

Treatment strategies for HL are stage-dependent. Early-stage disease is often managed with combination chemotherapy, such as the ABVD regimen (doxorubicin, bleomycin, vinblastine, dacarbazine), with or without involved-field radiotherapy [22]. Advanced-stage disease may require more intensive regimens like escalated BEACOPP (bleomycin, etoposide, doxorubicin, cyclophosphamide, vincristine, procarbazine, prednisone) [22]. In recent years, novel agents such as brentuximab vedotin, an anti-CD30 antibody-drug conjugate, and immune checkpoint inhibitors targeting PD-1 have shown efficacy, particularly in relapsed or refractory cases [23].

This complex postoperative case presented a diagnostic dilemma due to overlapping features of graft infection, HLH, drug reaction, and occult malignancy. Extensive imaging, microbiological work-up, H-score calculation, and empirical treatment with anakinra and steroids were employed to guide diagnosis while awaiting definitive histology. Ultimately, a bone marrow biopsy confirmed HL, allowing for targeted chemotherapy alongside continued monitoring for HLH and graft-related complications.

## Conclusions

This case highlights the importance of maintaining a broad differential when managing persistent systemic inflammation following surgery. HL should be considered when infection and autoimmune causes are excluded, especially in the presence of FDG-avid lymphadenopathy, cytopenias, and hyperferritinemia. Early hematology involvement and tissue diagnosis are key to avoiding delays in treatment.
